# The current progress and critical analysis of three-dimensional scanning and three-dimensional printing applications in breast surgery

**DOI:** 10.1093/bjsopen/zrab025

**Published:** 2021-04-08

**Authors:** S A Alshehri, S K Singh, A Mosahebi, D M Kalaskar

**Affiliations:** 1 UCL Division of Surgery & Interventional Science, Royal Free Hospital, London, UK; 2 Department of Surgery, King Faisal University, Al-Hofuf, Saudi Arabia; 3 Department of Burns & Plastic Surgery, Nottingham University Hospitals, Nottingham, UK; 4 Department of Plastic Surgery, Royal Free Hospitals NHS Trust, London, UK

## Abstract

**Background:**

Several attempts have been made to develop a tool capable of evaluating breast shape and volume to aid surgical planning and outcome assessment. More recently, newer technologies such as three-dimensional (3D) scanning and 3D printing have been applied in breast assessment. The aim of this study was to review the literature to assess the applicability of 3D scanning and 3D printing in breast surgery.

**Methods:**

A literature search was carried on PubMed, Google Scholar and OVID from January 2000 to December 2019 using the keywords ‘3D’, ‘Three-dimensional’, ‘Three/four dimensions’ and ‘Breast’.

**Results:**

A total of 6564 articles were identified initially; the abstracts of 1846 articles were scanned, and 81 articles met the inclusion criteria and were included in this review. Articles were reviewed and classified according to their aims, study subjects, the software and hardware used, main outcomes and major limitations.

**Conclusions:**

These technologies are fast and easy to use, however, high costs, long processing times and the need for training might limit their application. To incorporate these technologies into standard healthcare, their efficacy and effectiveness must be demonstrated through multiple and rigorous clinical trials.

## Teaser

3D scanning and 3D printing have been applied and investigated in breast surgery with promising potential. Although these technologies are fast and easy to use, increased costs, long processing time and the need for training might limit their application.

Needs further study

## Introduction

Conditions that disrupt the shape or symmetry of the breast may affect the individual's body image, quality-of-life scores, sexual functioning and psychological health^1–3^. The goals of breast surgery are to reconstruct the breast following partial or complete resection (often due to breast cancer), to aesthetically enhance breast shape or to correct a congenital or developmental deformity. Irrespective of its goal, there is an ongoing increase in the number of cosmetic and reconstructive surgeries performed worldwide^4^. Although these procedures are common, the rate of dissatisfaction is also high. In the UK, one in every four women was dissatisfied by her reconstructive surgery outcomes^5^. This could be attributed to several factors such as the communication gap between the expectations of the patient and the opinion of the surgeon or the lack of objective methods of shape and volume evaluation and outcome simulation.

**Fig. 1:**
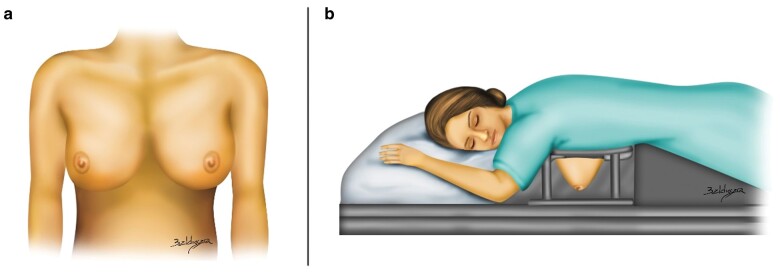
Comparison between breast shape in different positions **a** Breast shape as viewed in the normal sitting or standing position. **b** Breast shape distortion as the patient lies down prone during MRI scan. Illustration by Bruno Baldissara^14^.

Breast assessment is often done visually and depends on the skills and experience of the surgeon. Several methods of assessment have been investigated; however, these are often subjective, expensive, with limited accessibility, time consuming or simply inaccurate^6–13^. Breast shape also changes according to whether the patient is standing up or lying down in supine or prone positions. Although the breast shape is viewed normally in a standing or sitting position, most of the accurate imaging modalities such as CT or MRI are performed with the patient lying down in prone position. Therefore, in addition to their cost and limited accessibility, they cannot be used in breast shape assessment as the distortion produced by posture might affect surgical outcomes. *Figure 1* illustrates the difference in breast shape relative to body posture.

Although using MRI in breast assessment has many limitations including shape distortion, cost and clinical impracticality, it was reported as being both reliable and accurate in breast volume assessment. Xi and colleagues reviewed 276 articles from 1970–2013 and classified breast assessment methods depending on whether volume, shape or surface area was measured^15^. The settings, feasibility, reliability and reproducibility of results were compared. Despite their low accuracy, traditional devices such as measuring tapes, breast casts and Grossman-Rounder devices were cheaper and more accessible. However, MRI and 3D imaging devices were the most reliable. This is consistent with a review by Choppin and co-workers in which error quantification was used to determine the accuracy of breast volume assessment tools^16^. The most accurate method was MRI.

Currently, the objective evaluation of breast shape and volume, in addition to anticipating and simulating surgical outcomes, remain a research problem. With the advent of new technologies, such as 3D scanning and 3D printing, several authors have investigated its applicability to assess both breast shape and volume accurately. However, its clinical efficiency to improve objective measurements is not known.

3D surface scanning utilizes point cloud coordinates to construct an image of an object in 3D space. Although it was developed for industrial purposes, it is increasingly applied in medicine^17^^,^^18^. 3D scanning is done using either laser scanning devices, structured light devices or stereophotogrammetry. This technology can be incorporated into surgical planning, patient and staff education or outcome assessment. When compared to high-quality imaging techniques such as CT and MRI, 3D scanning is more convenient to the patient, with no position restriction or radiation exposure^19^^,^^20^.

The use of 3D scanning in breast assessment was first introduced by Galdino and colleagues in 2002^9^. Earlier studies lacked precision, used older or custom-made devices and recruited a small number of patients; however, they highlighted the ability of 3D scanning to evaluate the breast during surgical planning or outcomes assessment. Other studies compared the use of 3D surface imaging to the current methods of breast shape and volume assessment with varying outcomes^21^^,^^22^.

In the 1980s Charles Hull introduced the concept of 3D printing. This refers to the deposition of material in a layer-by-layer fashion to create a 3D object using data from a computer-aided design (CAD) file. Progress in this field resulted in shorter printing times and lower costs using numerous materials. CAD files are created using various software or obtained from CT, MRI or 3D scans. These files are then converted into a stereolithography file format and sent to the printer to be printed. The applications of 3D printing in medicine are countless and rapidly increasing. It can be used to print patient-specific implants and prostheses, biological scaffolds or anatomical models. The latter can be used for patient education, surgical planning and staff training^23^.

Recent systematic reviews reported that 3D printing increased the precision of preoperative planning and incision placement while reducing operative time and overall patient morbidity^23–26^. However, costs and the long processing time were the main limitations to applying this technology^23–26^.

The aim of this article is to assess the applicability of 3D scanning and 3D printing in breast surgery by reviewing the literature and classifying the studies based on study aims, main contribution and reported limitations. Extra elements were addressed, such as the hardware and software used to perform 3D scanning or 3D printing, in addition to the study subjects involved.

## Methods

###  

A literature search was carried on PubMed, Google Scholar and OVID from January 2000 to December 2019 using the keywords ‘3D’, ‘Three-dimensional’, ‘Three/four dimensions’ and ‘Breast’.

The inclusion criteria were:

- Articles that utilized 3D scanning devices, 3D printers or 3D simulation software to evaluate the shape and volume of the breast
- Articles that used the tools mentioned above to predict, evaluate or follow up surgical outcomes of breast surgery in any clinical or operative settings
- Articles that aimed to validate and calibrate 3D scanning devices, 3D printers or 3D simulation software using patients, volunteers or breast mannequins
- Articles that were in English or translated into English language
- Articles that were published in peer-reviewed journals

## Results

**Fig. 2:**
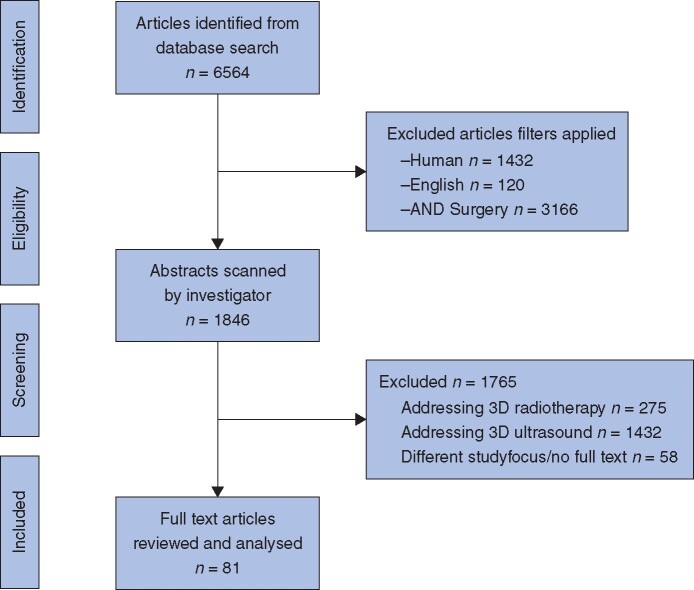
Flow diagram of articles selection based on defined inclusion and exclusion criteria used in this study

In total, 6564 articles were identified initially. Following inclusion criteria, abstracts of the final 1846 articles were scanned. Some 81 articles met the inclusion criteria and were included in this review (*Fig. 2*). Articles were later classified according to their aims, study subjects, the software and hardware used, main outcomes and major limitations. *TableSI* gives a summary of the articles included in this review.

### Study aims

**Fig. 3:**
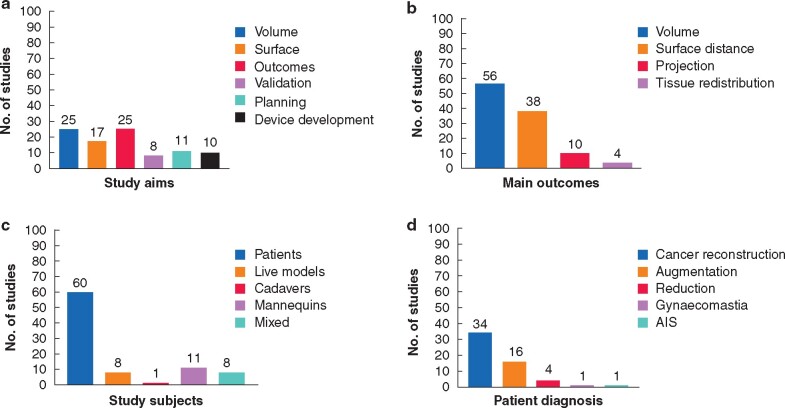
Bar diagrams representing the categorization of studies **a** Study aims: some studies have more than one aim. **b** Main outcomes: some studies measured more than one outcome. **c** Study subjects: mixed studies included both patients and mannequins. **d** Clinical diagnosis of the enrolled patients. AIS, adolescent idiopathic scoliosis

The studies had several different aims; some had more than one. The most common aims were to evaluate the accuracy of breast volume assessed via 3D scanning relative to other tools (25 studies, 31 per cent), and to monitor surgical outcomes and breast morphology changes (25 studies, 31 per cent). Seventeen (21 per cent) of the articles evaluated the accuracy of breast surface measurements using 3D scanning and 14 per cent (11 studies) addressed utilizing 3D scanning in presurgical planning, implant selection or simulation of the outcomes. Novel device development and validation were the focus of 12 per cent of the articles (10 studies) and the least investigated aim was the incorporation of 3D printing into patient surgery or the validation of 3D printed breast models (10 per cent, eight studies). *Figure 3a* displays the number of studies included relative to the aims.

### Main study outcomes

Assessment of breast volume was the most common outcome in 56 (69 per cent) of the reported studies. Evaluation of breast surface distances or symmetry as an outcome was the focus of 38 (47 per cent) of the studies. Breast projection was evaluated alongside these two outcomes in 10 (12 per cent) of the studies. Tissue migration or distribution was assessed in four (5 per cent) of the articles. *Figure 3b* presents the number of studies based on the main outcomes addressed.

### Study subjects

Human participants were recruited in 68 (84 per cent) of the included studies (*Fig. 3c*). Of these, eight studies recruited healthy volunteers and 60 studies recruited patients undergoing breast surgery. The most common diagnosis among the patients included was reconstruction following breast cancer (34 studies, 42 per cent) (*Fig. 3d*). Three of these articles focused on flap reconstruction after mastectomy. Sixteen studies (20 per cent) recruited patients having cosmetic breast augmentation and four (5 per cent) undergoing breast reduction. One study recruited patients with gynaecomastia and one study focused on adolescent idiopathic scoliosis patients. Three studies utilized breast mannequins alone and eight (10 per cent) utilized both human participants and mannequin models. One study was performed on a female human cadaver.

### Hardware devices

Most articles focused on the use of 3D scanners and cameras in breast evaluation. Only ten (12 per cent) addressed the applications of 3D printing either solely or in addition to 3D scanning. Four studies investigated simulation software and web-based assessment tools without using any hardware (5 per cent).

Several devices were used across the studies. Although the earliest reported device in the literature was the Rainbow 3D Camera^TM^ (Genex Technologies, Kensington), the most commonly used 3D scanning devices were the Vivid 910 device (Konika Minolta Sensing Inc., Osaka, Japan) and Vectra 3D imaging system (Canfield Scientific, Parsippany, NJ, USA). Overall, 12 studies (15 per cent) used the Vivid 910 device and 11 (14 per cent) used the Vectra 3D imaging system. In a further 10 studies (12 per cent), the devices were either custom-made or commercially unavailable or not described clearly. Earlier reports focused on the development and the investigation of one 3D scanning device or camera system. However, more recently there was a focus on comparing multiple devices with or without the incorporation of 3D printing.

### Software programs

Although 19 (23 per cent) of the studies relied on custom-made programs or did not specify the software used, the most commonly reported software was Geomagic studio (3D Systems Inc., Rock Hill, SC, USA), used in 19 (23.4 per cent) of the studies. 3D Rugle^©^ (Medic Engineering Co, Kyoto, Japan), Matlab^®^ (MathWorks Inc, Natick, MA, USA) and Breast Analysing tool (BAT^®^) were reported equally in four studies each (5 per cent each).

## Discussion

This study has demonstrated that 3D scanning and 3D printing have been applied for various purposes in breast evaluation and surgical management of breast disease. Due to the lack of objective tools capable of assessing breast shape and volume with high accuracy, there is a need to validate the applicability and accuracy of 3D scanning and 3D printing. It is not surprising, therefore, that most of the studies included in this review aimed to evaluate the accuracy of breast volume measurements, followed by monitoring surgical outcomes, in addition to morphology changes and assessment of breast surface measurements.

Although 3D printing has been known since the 1980s and authors have been investigating the applications of breast 3D scanning for the past two decades, only recently was 3D printing incorporated into their work. Investigating both techniques together can be an important step towards providing patients with patient-specific surgery based on accurate and objective measurements.

In terms of study subjects, most articles focused on human participants, specifically breast surgery patients and, among the patients recruited, the most common diagnosis was breast cancer. As these patients already undergo a wide range of imaging studies, incorporating 3D scanning and 3D printing into their management might be considered an extra unnecessary step. Considering the high dissatisfaction rates among post-reconstructive patients and the potential benefits that 3D scanning and 3D printing offer, adding this step might be justified. Some of these benefits were reported in a recent review by Diment and co-workers and included a reduction in operative time and overall morbidity in addition to improving surgeon–patient communication across various surgical specialties^23^; however, more research is needed in this area.

In addition to reconstructive breast surgery, the most common cosmetic procedures done globally are breast cosmetic surgeries^4^. Despite their popularity, the outcome of these procedures can result in high rates of patient dissatisfaction. This can be attributed in part to the gap between the surgeon’s opinion and the patient’s expectations and subsequently leads to an increase in the number of corrective procedures and overall morbidity. One quarter of the studies included in this review investigated using 3D scanning, 3D simulation and 3D printing within the context of cosmetic breast surgery. These technologies can be very useful in improving patient–surgeon communication in addition to providing them both with an approximation of the final cosmetic outcome.

In addition to applications in patient management, breast 3D scanning and 3D printing can be utilized in education and training of medical staff^23^^,^^24^. This is increasingly important when addressing disease presentations that are not frequently encountered. Given that the ethical prerequisites and privacy concerns are fulfilled, surgeons can globally exchange 3D scans of their patients and subsequently 3D print these scans into models for teaching and training purposes. This supplies the trainees with more tactile information and a better 3D perception of the breast instead of relying on flat two-dimensional images alone.

One of the objectives of this work was to review the types and trends of hardware devices used in the literature. Although a variety of devices were used for both 3D scanning and 3D printing, one of the difficulties encountered was that some studies did not provide a clear description of the hardware utilized or relied on custom-made devices with limited accessibility relative to the commercially available ones. This will make repeating these experiments by other researchers more challenging. Most studies did use commercially available devices with an early focus on the development and the investigation of one 3D scanning device or camera system, and more recently, a focus on comparing multiple devices with or without 3D printing.

There is a trend to move away from large devices that necessitate special set-up into smaller, portable and hand-held ones. For instance, the Microsoft Kinect game console (Microsoft, Redmond, Washington, USA) costs 108 USD and has a depth accuracy of 2 mm at 1 metre distance^27^. In addition, the Artec Eva scanner (Artec Europe, Luxembourg) retails at 19,800 USD and has a point accuracy of 0.1 mm at 1 metre distance^28^; and Structure Sensor (Occipital Inc., Colorado, USA) which has an accuracy of 0.5 mm at 40 cm and a price range of 449–695 USD^29^. Overall, this trend is expected due to the tendency to incorporate 3D scanning into patient care at clinical or operative settings, thus limiting the need for large set-ups and the number and duration of the visits required.

Several software programs were used to 3D scan, 3D print or simulate the breast surgery outcomes. Overall, the most used program was Geomagic studio by one quarter of the studies. A similar number utilized custom-made software programs or did not give clear description of the software used. This inconsistency in reporting makes repeating the experiments challenging and might explain why this technique has not been fully adopted.

Several studies reported that the cost of hardware, expensive software licensing, long processing time and the need to train staff were the main limitations to applying this technology. The 3D scanning devices vary in their specifications, accuracy and, consequently, cost. One study compared the costs of various devices and reported that to be within the range of 20 000–130 000 USD relative to 390 USD for CT scanning and 280–1400 USD for MRI scans^30^. Capturing and processing times were also compared and ranged from 0.001 to 5 seconds for image capturing and from 1 second to 15 minutes for processing^30^^,^^31^. Software post-processing of 3D scans might take a long time, but still less than the time required for CT imaging (90 minutes) and MRI imaging (13–30 minutes). Moreover, two studies aimed to compare low-cost 3D systems to more expensive ones and concluded that they could be applied clinically^32^^,^^33^. It is important that the accuracy and reproducibility of measurements is maintained as there is a move towards smaller, lower-cost, and portable devices. Software programs or online platforms that provide a simulation of breast surgery outcomes might be a feasible lower-cost option within the range of 4790–30 000 USD^30^ but assuring the security of these platforms is vital to maintain patient privacy.

Overall, when reviewing the studies, discrepancies relating to study reporting, external and internal validity were noticed. Some studies did not have a clear description of the aim and outcome measured. Moreover, patient characteristics, the interventions used, potential biases and confounders were not identified or addressed. There were no RCTs using 3D scanning or 3D printing in breast surgery applications. Most studies lacked sufficient power, did not include a control group, or did not compare 3D scanning and 3D printing to current imaging modalities such as MRI or CT scanning. To assess accurately the efficacy and effectiveness of 3D scanning and 3D printing in breast surgery it is important to conduct more clinical trials of sufficient power and test against a control group. The interventions used must be described clearly and several devices must be validated and investigated relative to MRI or CT scan.

## Supplementary material

Supplementary material is available at *BJS Open* online.

*Disclosure*. Authors declare they do not have any conflict of interest to declare. They have not received any funding from commercial companies for this work.

## Supplementary Material

zrab025_Supplementary_Data
