# Supplementary material for: The current progress and critical analysis of three-dimensional scanning and three-dimensional printing applications in breast surgery
Source: BJS Open. 2021 Apr 8;5(3):zrab025. doi: 10.1093/bjsopen/zrab025 (PMC8105620; doi:10.1093/bjsopen/zrab025)
Supplement: zrab025_Supplementary_Data [file zrab025_supplementary_data.docx]

| **NO** | **Article title & Author** | **Aims** | **Study Subjects** | **Device & Software** | **Main contribution** | **Limitations** | **Ref.** |
| --- | --- | --- | --- | --- | --- | --- | --- |
| 1 | ***Clinical Application of 3D Photography in Breast Surgery.***  *Galdino et al, 2002.* | Breast volume and projection assessment using 3D imaging. | 5 patients for breast augmentation, reduction, reconstruction, or correction of asymmetry. | Genex rainbow 3D camera. | 3D imaging is a valuable tool to plan breast surgery & monitor outcomes. | 1.The sample size is Small.  2. Obesity, dark skin, and ptotic breasts were difficult to scan. | [1] |
| 2 | ***Validating Three-Dimensional Imaging of the Breast.***  *Losken et al, 2005* | 1. To compare Volume of mastectomy specimen using 3D scanning & water displacement.  2. To compare nipple to notch distance using tape and 3D scanning. | 14 mastectomy patients. | 3dMD torso technology, 3dMD Analyze software. | 3D measurements of distance and volume were consistent and reproducible . However, they tend to be underestimated. | 1.The sample size is Small.  2. large breasts were difficult to scan.  3.size and posture variation were not addressed. | [2] |
| 3 | ***An Innovative Three-Dimensional Approach to Defining the Anatomical Changes Occurring after Short Scar-Medial Pedicle Reduction Mammaplasty.***  *Tepper et al, 2007.* | To monitor postoperative changes in anatomy, volume, tissue distribution, and projection using 3D scans. | 30 patients for breast reduction using short-scar medial pedicle. | Noncontact laser scanner V910, studio 9 Geomagic software. | 3D imaging was successfully used to monitor Preoperative and postoperative breast shape and volume changes. Using florescent light to decrease the shadows improves scan quality. | 1.The sample size is Small.  2. size and posture variation were not addressed. | [3] |
| 4 | ***Comparison Between Breast Volume Measurement Using 3D Surface Imaging and Classical Techniques.***  *Kovacs et al, 2007.* | To compare breast Volume obtained by 3D scanner , MRI, thermoplastic castings, and anthropomorphic measurements. | 6 female patients. | Minolta Vivid 910 3D linear laser scanner. | The most precise method was MRI followed by 3D scanner. | 1.The sample size is Small.  2. Size and posture variation were not addressed . | [4] |
| 5 | ***The Role of Three-Dimensional Scanning Technique in Evaluation of Breast Asymmetry in Breast Augmentation: A 100-Case Study.***  *Liu et al, 2010.* | To monitor postoperative changes in breast distances, width, projection, volume, and anterior chest wall using 3D scans. | 100 breast augmentation patients. | JRCB-D noncontact 3D scanner, Geomagic Studio 11. | 3D imaging was successfully used in analysing breast morphology and asymmetry during implant selection and surgical planning. |  | [5] |

**APPENDIX**

*Table 1: a summary of the articles included in this review*

| **NO** | **Article title & Author** | **Aims** | **Study Subjects** | **Device & Software** | **Main contribution** | **Limitations** | **Ref.** |
| --- | --- | --- | --- | --- | --- | --- | --- |
| 6 | ***Breast Volumetry Using a Three-Dimensional Surface Assessment Technique****.*  *Koch et al, 2011.* | To compare breast Volume obtained by 3D scanner to MRI. | 22 female patients. | Optical 3D sensor: BreastSCAN3D, software package (slim3D; 3D-Shape GmbH). | Breast volume can be assessed quickly and accurately using 3D imaging, but it is underestimated relative to MRI. | 1.The sample size is Small.  2. Size, BMI and posture variation were not addressed . | [6] |
| 7 | ***The Use of 3D Laser Imaging and a New Breast Replica Cast as a Method to Optimize Autologous Breast Reconstruction After Mastectomy.***  *Ahcan et al, 2012.* | To use reverse engineering in developing new breast replica cast (NBRC) for intraoperative flap shaping. | 12 patients undergoing unilateral autologous breast reconstruction with no ptosis (5 patients)  or grade I ptosis (7 patients). | The Lakos scanner. | Autologous breast reconstruction can be performed using 3D scanning and reverse engineering.  Survival rate of the Flap was 100% without corrective procedures. | 1.The sample size is Small.  2.Risk of flap ischemia during the NBRC shaping process.  3.Postoperative flap oedema was not addressed. | [7] |
| 8 | ***Accurate Assessment of Breast Volume: A Study Comparing the Volumetric Gold Standard (Direct Water Displacement Measurement of Mastectomy Specimen) With a 3D Laser Scanning Technique.***  *Yip et al, 2012.* | To compare preoperative specimen volume estimated by 3D scanning to intraoperative specimen volume estimated by water displacement. | 30 mastectomy patients. | Cyberware WBX Scanner, CySlice software. | volumes obtained using both methods were closely correlated. 3D scanners tend to slightly underestimate large breasts and overestimate smaller breasts. | 1.The sample size is Small.  2.Breast boundary definition was challenging.  3. Posture variation was not addressed . | [8] |
| 9 | ***Clinical Accuracy and Reproducibility of Portrait 3D Surgical Simulation Platform in Breast Augmentation.***  *Mailey et al, 2013.* | To use 3D surgical simulation platform in estimating actual and simulated preoperative volumes relative to postoperative volumes. | 22 breast augmentation patients. | Portrait 3D Surgical Simulation Platform. | Actual and simulated Breast volumes estimated using portrait 3D Surgical Simulation were accurate , reproducible with no statistically significant differences. | 1.The sample size is Small.  2.Breast boundary definition was challenging.  3. Short follow up period.  4.Body habitus , breast shape & size variations were not addressed. | [9] |
| 10 | ***Comparative Assessment of 3D Surface Scanning Systems in Breast Plastic and Reconstructive Surgery****.*  *Patete et al, 2013.* | 1. To compare breast scanning repeatability & accuracy using 2 commercially available hand-held 3D laser scanners and a prototype scanner. | Breast mannequin, 3 volunteers with varying breast sizes. | COMET5, Vivid 910 Konica, laser spot scanner SC3. | All scanners captured breast surface. Handheld laser scanners with motion compensation methods show promising potential in breast assessment. | 1.The sample size is Small.  2. Difficulties with scanning lateral breast and inframammary fold . 3.With repeated scanning, quality of scans was lower due to increased scanning time and posture change. | [10] |

| **NO** | **Article title & Author** | **Aims** | **Study Subjects** | **Device & Software** | **Main contribution** | **Limitations** | **Ref.** |
| --- | --- | --- | --- | --- | --- | --- | --- |
| 11 | ***3-D Analysis of Breast Morphology Changes After Inverted t-Scar and Vertical-Scar Reduction Mammaplasty Over 12 Months.***  *Eder et al, 2013.* | To use 3D imaging in evaluating soft tissue migration and oedema 12 months after inverted t-scar and vertical t-scar breast reduction. | 26 patients for inverted t-scar breast reduction and 22 patients for vertical-scar breast reduction. | Konica Minolta Vivid 910 3-D laser scanner, Geomagic Studio 12, & Geomagic Qualify 10 software. | 3D scanning successfully assessed changes in breast shape, volume, contour, soft tissue migration and surface area after breast reduction. | 1.The sample size is Small.  2. Resection weights between the groups was unequal. | [11] |
| 12 | ***3D Volumetric Analysis for Planning Breast Reconstructive Surgery.***  *Chae et al, 2014.* | To compare breast volume estimated using a reconstructed 3D scan and a 3D printed model to the volumes obtained using CT scan, MRI, and traditional methods . | 1 breast asymmetry patient following right breast free flap reconstruction. | Osirix software, Magics Software, Cube 2 printer. | Estimating breast volume by 3D scanning and 3D printing shortened operative time and was accurate and applicable in surgical planning. | 1. Posture taken during CT & MRI imaging distorted beast shape .  2.3D scanning and 3D printing is associated with increased costs , long processing time, and the need for staff training . | [12] |
| 13 | ***A Methodological Evaluation of Volumetric Measurement Techniques Including Three-Dimensional Imaging in Breast Surgery.***  *Hoeffelin et al, 2014* | To compare volume and notch to nipple distance using CT scan, Archimedes’ principle and 3D camera. | 9 female cadavers, 1 control prostheses, and 38 patients. | LifeViz 3D system, LifeViz DermaPix 3D software. | Measurements obtained from CT scan, Archimedes’ principle and 3D camera show good correlation. | 1.In-situ volume was underestimated.  3.Selecting patients was difficult. | [13] |
| 14 | ***A Prospective Study of Breast Dynamic Morphological Changes after Dual Plane Augmentation Mammaplasty with 3D Scanning Technique.***  *Ji et al, 2014.* | To use 3D scanning in monitoring breast changes after dual-plane augmentation . | 13 dual-plane breast augmentation patients. | JRCB-D noncontact 3D scanner, Geomagic Studio 11. | 3D scanning successfully assessed surgical outcomes and monitored dynamic changes. | 1.The sample size is Small.  2.Thie study utilized Only one surgical method. | [14] |
| 15 | ***Breast Volume Measurement by Mesh Projection Method Based on 3D Point Cloud Data.***  *Chen and wang 2014.* | To use 3D scanning in comparing breast volume from mesh projection of 3D point cloud data to water displacement volume. | 51 female volunteers. | TC2 3D scanner. | Volume estimation using this novel method was valid relative to the water displacement method. | 1.This method was developed for the purpose of clothes manufacturing and not clinical application. | [15] |

| **NO** | **Article title & Author** | **Aims** | **Study Subjects** | **Device & Software** | **Main contribution** | **Limitations** | **Ref.** |
| --- | --- | --- | --- | --- | --- | --- | --- |
| 16 | ***Breast Volume Measurement Using a Games Console Input Device.***  *Pohlmann et al, 2014* | To compare breast volume calculated from breast mould filled with liquid and measurements computed using Qiao formula to the volume obtained using Microsoft Kinect device. | Female torso model. | Games console input device – the Microsoft Kinect, Meshlab software, Matlab software. | There is good agreement between the volume obtained from Microsoft Kinect device and the volume obtained from liquid filled mould. | 1. Texture difference between model and human might affect results repeatability.  2. Shape & size variation were not addressed. | [16] |
| 17 | ***3D Surface Imaging of the Human Female Torso in Upright to Supine Positions.***  *Reece et al, 2015.* | 1.To evaluate the effect of tilt on breast morphology at different gravity loads. | 1 Rigid plastic mannequin & 5 female subjects. | 3dMD torso imaging system mounted to a bariatric tilted table. | Differences between supine and upright position correlated with BMI & bra cup-size and was more variable among the 5 subjects relative to the mannequin . | 1.The sample size is Small.  2.The use of a bariatric tilted table might not be clinically practical or comfortable to the patients. | [17] |
| 18 | ***A Comparative Study of Breast Surface Reconstruction for Aesthetic Outcome Assessment.***  *Lacher et al, 2017.* | To compare breast volume accuracy using hand-held Artec Eva scanner (for mannequin) and 3dMD (for patients) with low-cost RGBD Microsoft Kinect v1 and Kinect v2. | 1 Rigid mannequin & 12 breast surgery patients. | Artec Eva scanner, 3dMD, RGBD Microsoft Kinect v1 and Kinect v2. | Low-cost 3D scanning systems are clinically applicable in breast evaluation | 1.The sample size is Small.  2. Shape & size variation were not addressed. | [18] |
| 19 | ***Assessment of Breast Asymmetry in Adolescent Idiopathic Scoliosis Using an Automated 3D Body Surface Measurement Technique.***  *Ramsay et al, 2017.* | 1.To compare breast volume & asymmetry using 3D surface scans & MRI. | 30 AIS patients for evaluation of breast asymmetry. | Multi-head optical white-light scanning system Capturor II LF, Matlab software, FastRBF software. | There is strong correlation between volume obtained using 3D surface scanning and volume obtained using MRI. It is feasible and can be clinically applicable. | 1.The sample size is Small. | [19] |

| **NO** | **Article title & Author** | **Aims** | **Study Subjects** | **Device & Software** | **Main contribution** | **Limitations** | **Ref.** |
| --- | --- | --- | --- | --- | --- | --- | --- |
| 20 | ***Chances and Limitations of a Low-Cost Mobile 3D Scanner for Breast Imaging in Comparison to an Established 3D Photogrammetric System.***  *Koban et al, 2018* | To compare surface measurements and volume, using two types of 3D scanners before and after surgery. | 42 female breast surgery patients. | The portable sense TM handheld scanner, the VECTRA® XT device, Mirror software. | 1.There is high correlation with good repeatability of breast measurements using both devices.  2.Hand-Held 3D scanners are convenient and clinically applicable. | 1.The medical software during patient consultation was not available.  2.Colour-texture quality of the 3D images need to be improved. | [20] |
| 21 | ***Accuracy of Three Software Applications for Breast Volume Calculations from Three-Dimensional Surface Images.***  *Wesselius et al, 2018.* | To Evaluate 3D scanning accuracy through comparing preoperative 3D volumes with actual volumes of mastectomy specimens. | 26 mastectomy patients. | 3D BreAST, 3dMD Vultus, and VECTRA devices. | 1. Volumes obtained using 3D scanning devices and the volumes of weighed mastectomy specimens show no statistically significant differences.  2.There is a positive correlation between breast size and absolute error. | 1.The sample size is Small. | [21] |
| 22 | ***A Prospective Evaluation of Three-Dimensional Image Simulation: Patient-Reported Outcomes and Mammometrics in Primary Breast Augmentation.***  *Overschmidt et al, 2018* | Evaluation of patient-reported outcomes using (BREAST-Q) tool , breast mammometrics and preoperative simulation using Vectra 3D imaging software. | 100 female patients undergoing breast augmentation. | Vectra 3D imaging software, Vectra Sculptor package software. | 3D simulation was favoured by patients but did not significantly affect patient-reported outcomes or Mammometrics parameters. | 1.Patients were not distributed equally across study arms.  2.Possibility of selection bias as patients were offered the choice of group allocation.  3.Follow-up duration was short. | [22] |
| 23 | ***Applications and limitations of using patient-specific 3D printed moulds in autologous breast reconstruction.***  *Hummelink et al, 2018* | To apply 3D printed breast moulds during intraoperative flap shaping for delayed breast reconstruction. | 6 delayed breast reconstruction patients | 3dMD Body device and associated software, 3D software Autodesk 3ds Max, 3D printing software Cura. | 3D printed breast mould was successfully used for intraoperative flap shaping . | 1.The sample size is Small.  2. Shape & size variation were not addressed. | [23] |

| **NO** | **Article title & Author** | **Aims** | **Study Subjects** | **Device & Software** | **Main contribution** | **Limitations** | **Ref.** |
| --- | --- | --- | --- | --- | --- | --- | --- |
| 24 | ***3D Mammometric Changes in the Treatment of Idiopathic Gynecomastia.***  *Koban et al, 2019* | To use 3D Imaging in outcome assessment of bilateral subcutaneous mastectomy with waterjet assisted liposuction . | 30 male patients with Simon grade IIA to IIB breast enlargement and no ptosis. | Vectra 3D Imaging System, Mirror software. | 1.There is a lack of validated tools to assess Gynecomastia shape and volume in.  2.3D imaging is accurate but might be of little benefits to experienced surgeons. | 1.The sample size is Small. | [24] |
| 25 | ***Breast Shape Analysis with Curvature Estimates and Principal Component Analysis for Cosmetic and Reconstructive Breast Surgery.***  *Catanuto et al, 2019* | Novel method of breast assessment using infrared 3D scanner and principal  component analysis (PCA) . | 52 female volunteers. | The Structure Sensor 3D scanner. | Breast shape can be evaluated accurately using this novel method regardless of the operator’s skills. | 1.The system failed to record breast elasticity with motion.  2.Post processing was challenging. | [25] |
| 26 | ***The Effect of Respiration on Breast Measurement Using Three-Dimensional Breast Imaging.***  *Wang et al, 2019* | To assess differences in linear measurements and Preoperative breast volume change error (BVCE) relative to inspiration and expiration phases. | 13 patients for bilateral implant breast augmentation | JRCB-D 3D scanner, Geomagic studio 12 software. | There were no statistically significant differences in breast volume according to respiratory phase. | 1.The sample size is Small.  2. Shape , posture , ptosis & size variation were not addressed. | [26] |
| 27 | ***Bra band size Measurements Derived from Three Dimensional Scans are not Accurate in Women with Large, Ptotic Breasts.***  *McGhee et al, 2018* | To compare actual breast measurements (Bra-band size, Under-breast chest circumference (UBCC) and over-breast chest circumference (OBCC)), with 3D scan measurements. | 111 female volunteers. | Eva 3D Artec Scanner, Geomagic studio 12 software. | 3D scanning of large or ptotic breasts can be inaccurate and challenging. | 1.Distribution of participants into study groups was not equal.  2. Support during the scanning process affected breast shape but was not accounted for. | [27] |

| **NO** | **Article title & Author** | **Aims** | **Study Subjects** | **Device & Software** | **Main contribution** | **Limitations** | **Ref.** |
| --- | --- | --- | --- | --- | --- | --- | --- |
| 28 | ***Three-Dimensional Evaluation of Breast Augmentation and the Influence of Anatomic and Round Implants on Operative Breast Shape Changes.***  *Kovacs et al, 2012* | To evaluate breast measurements and volume changes using 3D imaging before and 6 months after surgery. | 17 patients for sub-pectoral breast augmentation with round implants and 10 patients for axillary approach with anatomical implants. | 3D laser scanner Vivid 910 Konica, Geomagic Studio 11 software. | 3D surface imaging is successful in tracking breast shape changes after augmentation mammaplasty. | 1.The sample size is Small.  2. limitations associated with retrospective study design. | [28] |
| 29 | ***Three-dimensional Scanning in Women with Large, Ptotic Breasts: Implications for Bra Cup Sizing and Design.***  *Coltman et al, 2017* | To investigate 3D scanning accuracy of large, ptotic breasts in 3 positions: standing with hands up, standing with hands on hip and lying prone positions. | 50 volunteers with large breasts >500 ml. | Eva 3D Artec Scanner, Geomagic studio 12 software. | 1.Breast volume in prone position was significantly larger than other positions.  2.Volume was underestimated in standing position.  3.Scanning of large ptotic breasts underestimated the volume. | 1. Volume measurements were variable.  2. Support during the scanning process affected breast shape but was not accounted for. | [29] |
| *30* | ***Magnetic Resonance Imaging Versus 3-Dimensional Laser Scanning for Breast Volume Assessment After Breast Reconstruction.***  *Howes et al, 2017* | To compare breast volume using 3D scanning with MRI before and 6 months after surgery. | 18 patients for autologous breast reconstruction with fat graft. | Cyberware whole-body 3D laser scanner, Cyslice software. | 3D laser scanning is easy to use and gave measurements that strongly correlated with MRI measurements. | 1.The sample size is Small.  2.Breast shape distortion because of gravity and position when undergoing the MRI scan. | [30] |
| *31* | ***Preoperative Implant Selection for Unilateral Breast Reconstruction using 3D Imaging with the Microsoft Kinect Sensor.***  *Pöhlmann et al, 2017* | To validate volume accuracy and reproducibility of the Kinetic recording system and whether it can predict the appropriate implant size. | 10 unilateral mastectomy patients with implant reconstruction. | Kinetic recording system by Microsoft, Meshlab software. | 3D imaging was used successfully to predict implant size preoperatively. | 1.The sample size is Small. | [31] |
| 32 | ***Validation of the Kinect Device as a New Portable Imaging System for Three-dimensional Breast Assessment.***  *Henseler et al, 2014* | To compare volume measured using Microsoft Kinetic system to the volume obtained using Arthur Morris device. | 9 silicone breast implants. | Kinect Recording System by Microsoft, Matlab software. | The kinetic recording device is able to assess breast volume with more accuracy and reproducibility relative to the Arthur Morris device. | 1.Results might differ if applied to humans.  2. Arthur Morris device to is not commonly used in practice. | [32] |

| **NO** | **Article title & Author** | **Aims** | **Study Subjects** | **Device & Software** | **Main contribution** | **Limitations** | **Ref.** |
| --- | --- | --- | --- | --- | --- | --- | --- |
| *33* | ***Development and Assessment of a Microsoft Kinect Based System for Imaging the Breast in Three Dimensions.***  *Wheat et al, 2014.* | To compare breast measurements using kinetic recording system with tape/calliper measurements. | Breast Mannequin. | Kinect Recording System by Microsoft,  Kinect for Windows SDK. | There is an acceptable agreement and high repeatability between the distances measured using the kinetic system and those measured manually. | 1. Manual Measurements are prone to error and incapable of measuring curvatures accurately. | [33] |
| *34* | ***Three-dimensional Evaluation of Breast Contour and Volume Changes Following Subpectoral Augmentation Mammaplasty over 6 Months.***  *Eder et al, 2011* | To evaluate breast morphology and volume changes, before and at 6 months after surgery. | 14 patients for subpectoral augmentation mammaplasty. | Konica Minolta Vivid 910, Geomagic Studio 11software, Geomagic Qualify 10 software. | 3D imaging successfully documented short- and long-term measurement changes as early as day 2-3 postoperatively up to 6 months ahead. | 1.The sample size is Small.  2.Variance in implant type and surgical approach. | [34] |
| 35 | ***Weighted regularize d statistical shape space projection for breast 3D model reconstruction.***  *Ruiz et al, 2018.* | Reconstruct and fitting of a 3D statistically based model of the breast (3DMM) using input from 2D pictures or 3D scans . | Anonymous database of 310 3D scans and 510 2D pictures of different breast surgery patients. | 3D morphable models (3DMM),  Structure Sensor by Occipital  and Crisalix’s software. | Introduction of the Weighted Regularized (WR) projection method to the 3DMM reconstruction of breast. | 1.Model reconstruction depends on mesh initialization which can be challenging.  2.Breast shape is always modified when applying statistical restriction to preserve its plausibility. | [35] |
| *36* | ***Validation of the Vectra XT three-dimensional imaging system for measuring breast volume and symmetry following oncological***  ***reconstruction.***  *O’Connell et al, 2018* | Validation of breast symmetry and volume measurements obtained using Vectra XT imaging system. | 6 breast phantom models with various volumes and 16 patients (11 mastectomy patients and 5 breast conserving surgery patients). | Vectra XT imaging system, Vectra Analysis Module (VAM) software. | The first validation of Vectra XT imaging system in breast symmetry and volume assessments. | 1.Small number of patients included.  2.Surgery type and body habitus were not addressed. | [36] |

| **NO** | **Article title & Author** | **Aims** | **Study Subjects** | **Device & Software** | **Main contribution** | **Limitations** | **Ref.** |
| --- | --- | --- | --- | --- | --- | --- | --- |
| *37* | ***Three-Dimensional Surface Imaging***  ***is an Effective Tool for Measuring Breast Volume: A Validation Study***  *Lee et al, 2016.*   \|  \| \| --- \| | To compare the volume of resected breast tissue calculated using weighing scale with the volume calculated using water displacement, MRI and 3D scanning. | 25 mastectomy patients undergoing breast reconstruction surgery. | Axis Three 3D scanner, AW4.6: Volume Viewer software, Axis Three imaging software. | Breast volume assessed using 3D scanning showed significant reliability when compared to volumes obtained using MRI, weighing scales and water displacement. | 1.Small number of patients included.  2.Determining breast borders on 3D scans lacked reproducibility. | [37] |
| *38* | ***Three-Dimensional Laser Imaging as a Valuable Tool for Specifying Changes in Breast Shape After Augmentation Mammaplasty***  *Esme et al, 2009* | To assess contour, shape, and volume intraoperatively and immediately after the placement of breast implants. | 31-year-old healthy patient undergoing Augmentation Mammaplasty. | FARO HS 880 TLS scanner, Cyclone 5.4 software package. | 3D TLS scanners can be used intraoperatively to objectively assess breast shape, contour and volume. | 1.Only one patient was enrolled.  2.Postoperative changes were not addressed or followed up. | [38] |
| 39 | ***Three-dimensional imaging, an important factor of decision in breast augmentation.***  *De Runz et al, 2018* | To investigate the attitude of breast augmentation patients towards their anticipated surgical outcomes using a web-based 3D simulator. | 38 patients undergoing consultation for breast augmentation. | Crisalix web-based 3D simulator. | The use of 3D simulation during consultation for breast augmentation was applicable and appreciated by the patients included. | 1.small number of study subjects.  2.Using 3D simulator prior to anticipate surgery outcomes can cause patient dissatisfaction or legal disputes. | [39] |
| *40* | ***Three-Dimensional Imaging Provides Valuable Clinical Data to Aid in Unilateral Tissue Expander-Implant Breast Reconstruction.***  *Tepper et al, 2007* | To investigate the applicability of 3D scanning within the context of tissue expander breast reconstruction. | 12 patients for immediate or delayed unilateral  tissue expander implant reconstruction. | Noncontact laser scanner V910, | 3D scanning is applicable in tissue expander breast reconstruction for choosing tissue expander size , implant volume and to monitor surgical outcomes (volume of expansion, shape, symmetry and the need for revision procedure). | 1.small number of study subjects. | [40] |

| **NO** | **Article title & Author** | **Aims** | **Study Subjects** | **Device & Software** | **Main contribution** | **Limitations** | **Ref.** |
| --- | --- | --- | --- | --- | --- | --- | --- |
| *41* | ***Three-dimensional Assessment of the Breast: Validation of a Novel, Simple and Inexpensive Scanning Process.***  *Oranges et al, 2019* | To investigate the Validity of using Structure Sensor by Occipital connected to iPad pro in breast assessment, relative to CT scan and other established 3D scanning devices. | Rigid plastic model of human torso. | Structure Sensor by Occipital connected to iPad pro, Vectra M5 Scanner, Artec Eva 3D scanner, Mimics Innovation Suite 20 software | 3D scans obtained using these novel inexpensive methods were reliable and valid in breast assessment. | 1.Study was applied using only one rigid plastic model and not accounting for breast size variation. | [41] |
| 42 | ***The potential role of three-dimensional surface imaging as a tool to evaluate aesthetic outcome after Breast Conserving Therapy (BCT) .***  *O’Connell et al, 2017* | To validate the use of 3D scanning in breast assessment among breast conserving therapy patients relative to their BREAST-Q questionnaire scores and panel assessment scores. | 200 patients who underwent unilateral breast conserving therapy within the past 6 years. | VECTRA XT device. | 3D surface scanning is objective and faster in assessing aesthetic breast outcomes following breast conserving therapy relative to panel assessment. |  | [42] |
| *43* | ***The Kinect Recording System for objective three- and four-dimensional breast assessment with image overlays.***  *Henseler et al, 2016* | To investigate the application of 3D and 4D scanning in breast assessment of female volunteers with regards to arm position and flap planning. | 6 female volunteers. | Kinect Recording System by Microsoft, Matlab software. | 3D surface scanning can be applied to obtain 3D and 4D scans of breast with satisfactory quality at different arm positions. Flaps can be planned using 3D polygonal mesh derived from scanning data. | 1.small number of study subjects. | [43] |
| *44* | ***The importance of the pose in three-dimensional imaging of the ptotic breast.***  *Henseler et al, 2013* | To investigate the validity and reproducibility of Custom-made positioning frame and multiple stereophotogrammetry 3D camera system in ptotic breast assessment. | 6 female volunteers. | Custom-made positioning frame, multiple stereophotogrammetry 3D camera system (8 digital cameras), dimensional imaging  software (Di3D), breast analysis tool (BAT) software, facial analysis tool software (FAT) and Clinical 3D software (C3D). | Ptotic breasts can be captured fully using multiple stereophotogrammetry 3D camera system and Custom-made positioning frame. | 1.small number of study subjects.  2.Positioning frame places the patient in uncomfortable position and causes breast shape to distort due to effect of gravity. | [44] |

| **NO** | **Article title & Author** | **Aims** | **Study Subjects** | **Device & Software** | **Main contribution** | **Limitations** | **Ref.** |
| --- | --- | --- | --- | --- | --- | --- | --- |
| 45 | ***Symmetrical Breast Reconstruction: Is There a Role for Three-Dimensional Digital***  ***Photography?***  *Nahabedian et al, 2003.* | To evaluate the applicability of 3D digital photography in assessing breast contour and volume symmetry after reconstruction. | 33 out of 382 cohort of breast reconstruction patients. | Genex Rainbow three-dimensional digital camera. | Utilizing 3D digital photography, patients undergoing autologous breast reconstructions had more initial and final symmetry relative to implant breast reconstruction. | 1.small number of study subjects.  2.Failure of the camera to capture breast volume beneath the cutaneous plane.  3.Mastectomy skin flaps might affect the accuracy of the volume calculated.  4.Diffuclties associated with imaging larger or ptotic breasts.  5.Diffuclties assuring the consistency in placing the landmarks to define breast borders. | [45] |
| *46* | ***Subjective versus objective assessment of breast reconstruction***  *Henseler* *et al, 2013.* | To evaluate the use of 3D camera in objective breast assessment following latissimus dorsi flap reconstruction relative to the subjective methods using 2D images. | 44 patients undergoing immediate unilateral breast reconstruction with extended latissimus dorsi flap. | Validated 3D multiple stereophotogrammetry imaging system and custom-made software. | 1.The first error study of Subjective breast assessment.  2.Objective breast assessment is more accurate and reproducible relative to subjective methods even if performed by a panel of experts. | 1.Unclear description of the 3D stereophotogrammetry imaging system and the software used.  2.small number of study subjects. | [46] |
| *47* | ***Re-defining pseudoptosis from a 3D perspective after short scar-medial pedicle reduction mammaplasty.***  *Small et al, 2010* | To evaluate breast volume, tissue distribution, projection and pseudoptosis following reduction mammaplasty using 3D scanner. | 15 patients undergoing medial pedicle reduction mammaplasty. | Noncontact laser scanner V910, Geomagic Studio 9. | 3D scanning can be used to track early and late post-operative changes in breast volume, tissue distribution, projection and pseudoptosis following short scar-medial pedicle reduction mammaplasty. | 1. Small number of study subjects.  2. Factors such as quality of skin, hormonal status, weight, patient, patient screening protocol, and surgical technique were not addressed. | [47] |
| *48* | ***Quantitative Analysis of the Reconstructed Breast Using a***  ***3-Dimensional Laser Light Scanner.***  *Isogai et al, 2006.* | To assess breast shape, volume and symmetry quantitatively following breast reconstruction using 3D scanning device. | 51 breast cancer patients undergoing various types of breast reconstruction procedures. | Laser Light Scanner,  Voxelan NKV-300DM, 3d-Rugle software. | 3D scanning can be used to evaluate breast shape, volume and symmetry following reconstruction in breast cancer patients. | 1. Small number of study subjects in each group.  2. Data defects due to technical issues. | [48] |

| **NO** | **Article title & Author** | **Aims** | **Study Subjects** | **Device & Software** | **Main contribution** | **Limitations** | **Ref.** |
| --- | --- | --- | --- | --- | --- | --- | --- |
| *49* | ***Preoperative implant selection for two stage breast reconstruction with 3D imaging***  Szychta et al, 2014 | To compare the repeatability and accuracy of breast measurements (height, width, projection and volume) obtained using measuring tape relative to measurements obtained using casting and 3D scanning. | 50 patients undergoing unilateral two-stage breast reconstruction with implants. | Customized 3D camera, Antroposcan3D software (developed by author). | 3D scanning can be precise when selecting implant for breast reconstruction relative to anthropometric measurements and thermoplastic casting. | Small number of study subjects. | [49] |
| *50* | ***Objective evaluation of the latissimus dorsi flap for breast reconstruction using three-dimensional***  ***Imaging.***  *Henseler et al, 2012.* | To evaluate breast shape and volume using 3D multiple camera  Imaging system following unilateral breast reconstruction with latissimus dorsi flap  . | 44 breast cancer patients undergoing immediate unilateral reconstruction with latissimus dorsi muscle flap. | Custom-made positioning frame, multiple stereophotogrammetry 3D camera system,  Breast analysis tool (BAT) software, Clinical 3D software (C3D),  Dimensional Imaging software (Di3D). | 3D imaging system was used to demonstrate that unilateral reconstruction using latissimus dorsi flap does not achieve symmetry with the contralateral breast. | 1. Small number of study subjects.  2. Inaccurate positioning of landmarks on the breast affects the accuracy of measurement. | [50] |
| *51* | ***Objective breast symmetry evaluation using 3-D surface imaging.***  *Eder et al, 2012* | To assess breast symmetry and contour difference-using 3D scanning relative to 2D measurements obtained using BCCT.core software. | 2 dummy models  10 test subjects  30 patients undergoing breast augmentation or breast reduction  23 patients undergoing secondary free TRAM reconstruction. | Noncontact laser scanner V910, Geomagic Studio 11 Qualify 10 software, BCCT.core software. | 3D imaging can be used in the objective assessment and documentation of breast asymmetry. | Small number of study subjects. | [51] |
| *52* | ***Non-contact imaging of breast***  ***surface for breast surgical planning.***  *Tong, et al, 2019* | To scan breast with the patient in supine position through the development of a non-contact 3D imaging system. | 3D printed full-size breast model. | Two HP 3D structured light scanner pro  S3 mounted on articulating arm and a cart, HP 3D Scan Pro software, Meshlab software, CloudCompare software. | The developed 3D scanning system can be used to scan the 3D printed breast model in supine position with submillimetre accuracy (0.2 mm) and good resolution (0.1 mm) | 1. The model used is rigid and does not account for human breast variations in size, skin and texture.  2. Gravity effect on the breast was not addressed. | [52] |

| **NO** | **Article title & Author** | **Aims** | **Study Subjects** | **Device & Software** | **Main contribution** | **Limitations** | **Ref.** |
| --- | --- | --- | --- | --- | --- | --- | --- |
| *53* | ***New Aspects of Breast Volume Measurement Using 3-Dimensional Surface Imaging***  *Kovacs et al, 2006.* | To evaluate the accuracy and precision of breast measurements obtained using 3D scanning by 5 different observers relative to MRI measurements. | 2 human breast models.  6 test subjects.  10 patients undergoing breast surgery. | Minolta Vivid 910 3D linear laser scanner, Geomagic Studio7 software, HepaVision Software. Raindrop Geomagic Qualify 7 Software. | 1. Breast volume assessed using 3D scanning shows good accuracy with high correlation to MRI measurements.  2.3D scanning is user independent.  3. The chest wall can be demarcated using appropriate software and shows high agreement with MRI. | 1. Small number of study subjects.  2. Difficulties in defining breast borders.  3. Factors such as anatomic limitations, post-operative swelling, and tissue redistribution are not addressed.  4. Difficulties when scanning large ptotic breasts, obese patients or patients with chest wall deformities. | [53] |
| *54* | ***An innovative method of planning and***  ***displaying flap volume in DIEP flap***  ***breast reconstructions.***  *Hummelink et al, 2017.* | To investigate the applicability of virtual flap planning using projection method, 3D stereophotogrammetry and computed tomography angiography (CTA) in patients undergoing DIEP flap breast reconstruction. | 6 patients undergoing DIEP flap breast reconstruction. | 3dMD Body scanning device, 3ds Max software, VitreaAdvanced fX workstation software, | The shape and volume of the DIEP can be planned virtually using projection method, 3D stereophotogrammetry and computed tomography angiography (CTA) prior to breast reconstruction surgery. | Small number of study subjects. | [54] |
| *55* | ***Multi‑view stereophotogrammetry for post‑mastectomy breast reconstruction.***  *Ju et al, 2016.* | To compare breast volume obtained using a custom-made 3D stereophotogrammetry device with volumes obtained using water displacement method. | 9 plaster breast models | a custom-made Multi-view 3D photogrammetry imaging system, | Breast volume measured using the custom-made Multi-view 3D photogrammetry imaging system was more reliable than volumes obtained using water displacement method. | 1. Study was conducted using a custom-made 3D photogrammetry device and breast plaster models which might not be feasible to obtain or applicable on human breast. | [55] |
| *56* | ***Measurement of breast volume using body scan technology (computer-aided anthropometry).***  *Veitch et al, 2012.* | To validate the assessment of breast volume using 3D scanning (computer-aided anthropometric) among mastectomy patients. | 1 Large-size breast model  30 mastectomy patients with obesity, ptosis or size C and larger breast. | Cyberware WBX, metamorphosis software. | Breast volume obtained in this difficult patient group (obesity, ptosis, large breast size) using 3D scanning highly correlated with the gold standard method of volume assessment using water displacement. | 1. Inability to define breast border by palpitation due to the rigidity of the model unlike the human breast.  2. Overestimation of breast volume obtained through 3D scanning due to the inclusion of breast skin.  3. Only breast volume was addressed without evaluating shape, size or position. | [56] |

| **NO** | **Article title & Author** | **Aims** | **Study Subjects** | **Device & Software** | **Main contribution** | **Limitations** | **Ref.** |
| --- | --- | --- | --- | --- | --- | --- | --- |
| *57* | ***Is Unilateral Implant or Autologous Breast Reconstruction Better in Obtaining Breast Symmetry?***  *Cohen et al, 2016* | To use 3D scanning in the evaluation of breast shape, size, and projection following unilateral breast reconstruction using implants relative to autologous breast reconstruction using flap. | 64 post-mastectomy patients undergoing breast reconstruction with implants (n=34) or autologous flap (n=30). | Vectra scanner, Geomagic software. | 3D scanning can be used to objectively compare the surgical outcomes of unilateral breast reconstruction using implants relative to autologous flap reconstruction. | 1.Short follow-up period (1 year)  2. Patient satisfaction, sensibility or naturalness is not addressed when using 3D scanning. | [57] |
| *58* | ***Investigation into variation and errors of a three-dimensional breast imaging system using multiple stereo cameras.***  *Henseler et al, 2012.* | To evaluate the variations and errors in breast volume assessment when using a custom-made 3D stereophotogrammetry system and its relation to breast size, relative to volume obtained using water displacement method. | 9 plaster breast model  6 live volunteers. | Custom-made positioning frame, Multiple stereophotogrammetry 3D camera system, breast analysis tool (BAT) software, dimensional imaging software (Di3D) and Clinical 3D software (C3D). | 3D scanning using Multiple stereophotogrammetry 3D system is applicable in evaluating the relation between breast size and the errors and variations in breast volume assessment. | 1. Small number of study subjects.  2. Study was conducted using a custom-made 3D photogrammetry device which might not be feasible to obtain. | [58] |
| *59* | ***Intraoperative application of three-dimensional imaging for breast surgery.***  *Tanabe et al, 2005* | To analyze breast volume and contour intraoperatively using 3D scanning. | 2 patients undergoing breast reconstruction using flap or implants. | Vivid 300 3D scanner, Rugle 3 software, | 3D scanning device can be applied safely intraoperatively to analyze breast volume and contour. | 1. Small number of study subjects.  2. Large or ptotic breasts can be difficult to scan.  3. intraoperative swelling or postoperative volume change were not addressed. | [59] |
| *60* | ***Evaluation of volume and shape of breasts: Comparison between traditional and three-dimensional techniques.***  *Eriksen et al, 2011.* | To evaluate the accuracy and reproducibility of breast shape and volume obtained using 3D techniques with the shape and volume obtained using traditional methods (plastic cups, thermoplastic casts and MRI) pre- and post-operatively. | 12 patients undergoing primary breast reconstruction with implants. | VIVID 910 laser scanner, Geomagic software, MeX software and 3D stereophotogrammetry camera system. | Unlike breast shape measurement using 3D scanning, the accuracy of volume measurements obtained using 3D scanning is questionable relative to other methods. | 1. Small number of study subjects.  2. Difficulties in recognizing breast border when using MRI resulted in overestimation of breast volume. | [60] |

| **NO** | **Article title & Author** | **Aims** | **Study Subjects** | **Device & Software** | **Main contribution** | **Limitations** | **Ref.** |
| --- | --- | --- | --- | --- | --- | --- | --- |
| *61* | ***Enhancing breast projection in autologous reconstruction using the St Andrew’s coning technique and 3D volumetric analysis.***  *Chae et al, 2017.* | To evaluate the applicability of 3D scanning and 3D printing of a mirror image of the contralateral breast in guiding the planning of delayed unilateral breast reconstruction with DIEP flap. | 3 patients undergoing delayed unilateral breast reconstruction with DIEP flap. | VECTRA XR scanner, Magics software, MakerBot Desktop software, MakerBot Z18 3D printer | 3D scanning and 3D printing of the contralateral breast can be applied together with the St Andrew’s conning technique to enhance the aesthetical outcomes of unilateral breast reconstruction using the DIEP flap. | 1. Small number of study subjects.  2. Short follow-up period.  3. None of the patients required a secondary procedure to correct the contralateral breast, thus this technique was not investigated under these circumstances. | [61] |
| *62* | ***Does Respiration Influence Breast Volumetric Change Measurement with the Three-Dimensional Scanning Technique?***  *Liu et al, 2014* | To investigate the effect of respiration phase on the accuracy of breast volume measured using 3D scanning. | 10 patients undergoing augmentation mammaplasty. | JRCB-D noncontact 3D scanner, Geomagic Studio 12 software | Breast volume measurements obtained using 3D scanning varies according to the respiration phase. Position and respiration phase must be controlled to reduce this variation. | Small number of study subjects. | [62] |
| *63* | ***Computation of breast ptosis from 3D surface scans of the female torso.***  *Li et al, 2016* | To investigate the applicability of 3D scanning in the objective assessment of breast ptosis relative to subjective methods. | 145 patients undergoing breast reconstruction surgery.  5 volunteers. | DSP800 system, 3dMDTorso system, | 3D evaluation of breast ptosis , orientation and curvature through a distance-independent approach is highly accurate and precise relative to expert rating, anthropometry, or 2D photogrammetry. | 1.No description of the software used.  2.Patient position , type and timing of surgery were not addressed. | [63] |
| *64* | ***The role of three‐dimensional printing in the surgical management of breast cancer.***  *Santiago et al, 2019* | To explore the incorporation of a patient‐specific 3D printed model in breast cancer management. | 1 breast cancer patient undergoing mastectomy. | FORM 2 printer, InPrint 3.0 software. | Using patient‐specific 3D printed model in breast cancer management can help in patient education and decision-making during consultation. | 1. Study is limited to one patient. | [64] |

| **NO** | **Article title & Author** | **Aims** | **Study Subjects** | **Device & Software** | **Main contribution** | **Limitations** | **Ref.** |
| --- | --- | --- | --- | --- | --- | --- | --- |
| *65* | ***Customized Planning of Augmentation Mammaplasty with Silicon Implants Using Three-Dimensional Optical Body Scans and Biomechanical Modelling of Soft Tissue Outcome.***  *Gladilin et al, 2011.* | To investigate the application of a customized 3D body scanning method in objective assessment of augmentation mammaplasty and simulation of postoperative results. | 3 out of 39 patients undergoing augmentation mammaplasty with implants. | Vectra scanner. | 3D body scanning can be used in the objective assessment of augmentation mammaplasty to guide implants selection and simulate the post-surgical outcomes. | 1.Small number of study subjects.  2.Diffuctiles associated with scanning & simulating patients with large or ptotic breasts.  3.Unclear description of the software used. | [65] |
| *66* | ***Development and Implementation of a Web-Enabled 3D Consultation Tool for Breast Augmentation Surgery Based on 3D-Image Reconstruction of 2D Pictures.***  *Ciechomski et al, 2012.* | To develop a personalized web-based breast reconstruction simulation tool and validate it in relation to 2D photographs and 3D scanning device. | 11 anonymised clinical cases for surface reconstructions.  4 anonymised clinical cases for postoperative simulations. | Escan3D device , Amira 5.3.3 software. | The investigated web-based breast reconstruction simulation tool can be applied successfully in breast augmentation clinical scenarios. | 1.Small number of study subjects. | [66] |
| *67* | ***Correlation of Prediction and Actual Outcome of Three-Dimensional Simulation in Breast Augmentation Using a Cloud-Based Program.***  *Vorstenbosch and Islur, 2017.* | To compare simulated aesthetic outcomes (breast shape and volume) using a 3D simulation software with real postoperative outcomes. | 20 patients undergoing breast augmentation. | Crisalix software | 3D simulation can anticipate post-operative breast augmentation outcomes. This ability needs to be improved for ptotic and tuberous breasts. | 1.Small number of study subjects.  2.Patients received round implants in sub-glandular plane only. Other implant types or insertion planes were not addressed. | [67] |
| *68* | ***Accurate Assessment of Breast Volume by Computed Tomography Using Three-dimensional Imaging Device.***  *Fujii et al, 2012.* | To evaluate the use of computed tomography (CT) using a three-dimensional device in the assessment of breast volume relative to the intraoperative specimen volume. | 10 breast cancer patients undergoing mastectomy with immediate breast reconstruction. | WS ziostation 3d | Breast volume calculated using the CT 3D method shows a strong correlation relative to the volume calculated from surgical specimen as control. | 1. Small number of study subjects.  2. Retrospective collection of data. | [68] |
| *69* | ***DIEP Flap Breast Reconstruction Using 3-dimensional Surface Imaging and a Printed Mold.***  *Tomita et al, 2015.* | To evaluate the application of 3D scanning and 3D printing in estimating the volume of DIEP flap needed in unilateral breast reconstruction. | 11 patients undergoing immediate or delayed unilateral breast reconstructions with deep inferior epigastric artery perforator (DIEP) flaps | David Structured Light Scanner SLS-1, Breast-Rugle 3D image data analysis software, public domain image software, MakerBot Replicator 2x 3D printer. | 3D printed mold obtained from 3D images of the contralateral breast can be applied safely and successfully in planning breast reconstructive procedure using DIEP flap. | 1. Small number of study subjects.  2. Errors in estimating breast volume due to the inability of the 3D scanner to determine Skin envelope thickness and chest wall shape.  3. Slight overestimation of the reconstructed breast volume. | [69] |

| **NO** | **Article title & Author** | **Aims** | **Study Subjects** | **Device & Software** | **Main contribution** | **Limitations** | **Ref.** |
| --- | --- | --- | --- | --- | --- | --- | --- |
| *70* | ***DIEP Flap Breast Reconstruction in Patients with Breast Ptosis: 2-Stage Reconstruction Using 3-Dimensional Surface Imaging and a Printed Mold.*** *Tomita et al, 2017* | To evaluate the use of 3D scanning and 3D printing to achieve breast symmetry in patients undergoing 2 stage DIEP flap breast reconstruction with TE and contralateral mastopexy. | 8 breast cancer patients with ptotic breasts underwent 2 stage DIEP flap breast reconstruction with TE and contralateral mastopexy. | David Structured Light Scanner SLS-1, Breast-Rugle 3D image data analysis software, MakerBot Replicator 2x 3D printer. | 3D printed mold obtained from 3D images of the contralateral breast can be applied safely and successfully in planning 2 stage breast reconstructive procedure using DIEP flap in patients with ptotic breasts. | 1. Small number of study subjects.  2. Patients had to undergo 2 surgeries. | [70] |
| *71* | ***What Makes a Difference? Three-Dimensional Morphological Study of Parameters that Determine Breast Aesthetics.*** *Chen et al, 2020* | To investigate which breast parameter is more important aesthetically relative to other parameters within the context of patient satisfaction and breast aesthetics | 179 unilateral breasts of patients undergoing breast cosmetic surgery. | JRCB-D noncontact 3D scanner, Geomagic Studio 10 software | With the aid of 3D scanning, several parameters were investigated to determine aesthetic priority. Breast projection and vertical distance from nipple to lower breast fold were the most important parameters. Breast width did not differ significantly between high-satisfaction and low-satisfaction groups. | 1. Study was limited to cosmetic surgery patients . 2. Unequal distribution of patients across study groups. 3. The scale used to obtain score was not a validated scale. | [71] |
| *72* | ***Semi-Automated Delineation of Breast Cancer Tumors and Subsequent Materialization Using Three-Dimensional Printing (Rapid Prototyping).*** *Schulz-Wendtland et al, 2017* | To develop and assess the accuracy of 3D printed models of breast tumors obtained through MRI imaging and compare it to tumor volumes assessed using different imaging modalities (MRI,  DBT, 3D US). | 5 invasive breast cancer patients. | MeVisLab framework software, Alphacam Dimension SST1200 3D printer, | 3D printing can be used successfully to print tumor models through semi-automated delineation of the MRI scan. This can be applied pre-operatively to enhance the surgeon’s understanding of the tumor’s shape and size in order to decrease the need for re-excision. | Small number of study subjects. | [72] |
| *73* | ***Reproducing two-dimensional mammograms with three-dimensional printed phantoms.***  *Badal et al, 2018* | To validate the 3D printed phantom breast models obtained from 2D mammograms relative to the original mammograms. | Three 3D printed breast phantoms | Mammoreplicator software, inkjet-based Objet260 Connex3 printer. | Patient -specific breast phantoms can be obtained from mammograms using 3D printing with good similarity between the 3d printed model and the original mammogram. | 1. Small number of printed models. 2. Uncertainties were encountered due to using different mammography systems from different manufacturers or due to difference in the acquisition source-to-phantom distances. | [73] |

| **NO** | **Article title & Author** | **Aims** | **Study Subjects** | **Device & Software** | **Main contribution** | **Limitations** | **Ref.** |
| --- | --- | --- | --- | --- | --- | --- | --- |
| *74* | ***Preoperative breast volume evaluation of one-stage immediate breast reconstruction using three-dimensional surface imaging and a printed mold.*** *Chen et al, 2019.* | 1.To compare the accuracy of breast volume obtained using 3D scanning with the volume obtained using MRI and water displacement method.  2.To develop a 3D printed mold and use it during one-stage immediate breast reconstruction. | 19 Patients undergoing one-stage immediate breast reconstruction following mastectomy. | Artec Eva 3D scanner, Artec Studio 12 Professional software, Meshmixer software, Slicer4 version 4.11.0 software, UltimakerCura 3.0.3 software, CR-10s 3D printer. | 1. Breast volume measured using 3D scanning is comparable to the accuracy and reproducibility of the volume obtained using MRI.  2.3D printed mold can be applied successfully in one-stage autologous breast reconstruction and provides better symmetry and aesthetic outcomes. | 1. Small number of study subjects. 2. Variability of breast size and shape were not addressed. 3. Difficulties in defining breast borders in high BMI patients. 4. The presence of NAC or skin in the specimen affects the accuracy of volume measurement using water displacement method. 5. Difficulties determining chest wall. | [74] |
| *75* | ***New Technologies for the Assessment of Breast Surgical Outcomes.***  *Catanuto et al 2009* | To investigate the accuracy of both static and dynamic breast shape evaluation using a prototypical 3D/4D custom-made laser scanner. | 7 volunteers including those who underwent reconstructive breast surgery, cosmetic breast surgery or didn’t undergo either. | A custom made 3-D/ 4D handheld laser scanner, Breast Shape Analyzer software. | The aforementioned custom made 3-D/ 4D handheld laser scanner is reliable in assessing breast surface during both static and dynamic positions. | 1. Small number of study subjects.  2. Long acquisition time (10 minutes).  3. Scanning can be an extra step in the management of cancer patients.  4. Technical and clinical validation of the device is still required. | [75] |
| *76* | ***MRI-based 3D-printed surgical guides for breast cancer patients who received neoadjuvant chemotherapy.***  *Ko et al 2019.* | To investigate the use of patient specific 3D printed surgical guides of the breast and tumors in the surgical management of breast cancer. | 5 breast cancer patients receiving neoadjuvant chemotherapy and  Undergoing breast conserving surgery. | Connex3 Objet500 3D printer. | Patient specific 3D printed surgical guides obtained from MRI images can be successfully applied to mark and confirm the location of the tumour during breast conserving surgery. | 1. Pilot study with small number of study subjects.  2. Specifications and details of the software used were not included. | [76] |
| *77* | ***Menstrual Cycle-Related Fluctuations in Breast Volume Measured Using Three-Dimensional Imaging: Implications for Volumetric Evaluation in Breast Augmentation.***  *Wang et al 2019.* | To investigate the correlation between menstrual cycle and breast volume measured using 3D scanning. | 13 patients with regular menstrual cycles. | JRCB-D 3D scanning device, Geomagic Studio 12 software. | There is a statistically significant difference in breast volume before and after menstruation and this must be considered when planning breast surgery. | 1. Small number of study subjects.  2. All study subjects had relatively small breast size, the effect of ptosis or large size was not addressed.  3. Hormone levels were not measured. | [77] |

| **NO** | **Article title & Author** | **Aims** | **Study Subjects** | **Device & Software** | **Main contribution** | **Limitations** | **Ref.** |
| --- | --- | --- | --- | --- | --- | --- | --- |
| *78* | ***Measuring Volumetric Change After Augmentation Mammaplasty Using a Three-Dimensional Scanning Technique: An Innovative Method .*** *Liu et al 2012* | To evaluate the accuracy, of breast volume measured prior to augmentation mammaplasty using 3D scanning and postoperative volume constructed via 3D simulation relative to the true volume change. | 5 patients undergoing breast augmentation. | Geomagic Studio 11 Software. | 3D scanning can be used to simulate post-operative volume change in augmentation mammaplasty patients with excellent accuracy and repeatability. | 1. Small number of study subjects.  2. Variations in size, ptosis, and procedure type were not addressed. | [78] |
| *79* | ***A Prospective Randomized Study Comparing Two Different Expander Approaches in Implant-Based Breast Reconstruction: One Stage versus Two Stages.*** *Eriksen et al 2012* | To assess post-operative breast shape and volume using 3D scanning relative to traditional methods: Plastic cups, plastic casts, and 2D imaging techniques.  To evaluate the aesthetic outcomes by obtaining patient opinion, quality of life survey scores and panel of experts’ assessment.  To determine which procedure had lower number of operations needed to achieve patient satisfaction between the 2 groups (one-stage; round implants) vs. (two-stage; crescent implant). | 40 (out of 70) breast cancer patients undergoing one-stage or two-stage implant-based reconstruction with either round or crescent expander implants. | Minolta Vivid 910 3D linear laser scanner, Geomagic software. | 3D scanning can be used to subjectively evaluate breast reconstruction. The success of round permanent expander implants in a one-stage procedure was less than the two-stage procedure using crescent implants. | 1. Small number of study subjects.  2. Patients with obesity were excluded although weight and large breast size influences breast evaluation.  3. The permanent anatomically shaped expander (Becker 35) was not investigated. | [79] |
| *80* | ***A Patient-Specific 3D-Printed Form Accurately Transfers Supine MRI-Derived Tumor Localization Information to Guide Breast Conserving Surgery.*** *Barth Jr et al 2017* | To develop a patient specific device to aid in the localization of non-palpable breast tumor using data from supine MRI and 3D printing techniques. | 19 breast cancer patients undergoing partial mastectomy. | Go!Scan 3D scanner device, | 3D printing can be applied safely and accurately to develop patient specific device (Breast Cancer Locator (BCL)) to localize breast cancer using data from supine MRI. | 1.Small number of study subjects.  2. Details of the 3D printer used, and the software applied were not discussed. | [80] |
| *81* | ***Three-Dimensional Imaging and Breast Measurements: How Predictable Are We?*** *Steen et al, 2017* | To determine the accuracy 3D imaging in breast surface measurement relative to direct measurements obtained by two raters. | 28 patients undergoing augmentation mammaplasty. | Vectra XT 3D Imaging System , Vectra XT 3D software. | Measurements obtained using 3D scanning have an acceptable mean difference relative to the measurements obtained directly. However, this varies according to the measured plane. | 1. Small number of study subjects.  2.Diffuclties encountered when measuring the N-IMF distance and defining breast borders.  3.Error in direct measurements cannot be totally ruled out. | [81] |

**References**

1. Galdino, G.M., et al., *Clinical applications of three-dimensional photography in breast surgery.* Plast Reconstr Surg, 2002. **110**(1): p. 58-70.

2. Losken, A., et al., *Validating three-dimensional imaging of the breast.* Ann Plast Surg, 2005. **54**(5): p. 471-6; discussion 477-8.

3. Tepper, O.M., et al., *An innovative three-dimensional approach to defining the anatomical changes occurring after short scar-medial pedicle reduction mammaplasty.* Plast Reconstr Surg, 2008. **121**(6): p. 1875-85.

4. Kovacs, L., et al., *Comparison between breast volume measurement using 3D surface imaging and classical techniques.* Breast, 2007. **16**(2): p. 137-45.

5. Liu, C., et al., *The role of three-dimensional scanning technique in evaluation of breast asymmetry in breast augmentation: a 100-case study.* Plast Reconstr Surg, 2010. **126**(6): p. 2125-32.

6. Koch, M.C., et al., *Breast volumetry using a three-dimensional surface assessment technique.* Aesthetic Plast Surg, 2011. **35**(5): p. 847-55.

7. Ahcan, U., et al., *The use of 3D laser imaging and a new breast replica cast as a method to optimize autologous breast reconstruction after mastectomy.* Breast, 2012. **21**(2): p. 183-9.

8. Yip, J.M., et al., *Accurate assessment of breast volume: a study comparing the volumetric gold standard (direct water displacement measurement of mastectomy specimen) with a 3D laser scanning technique.* Ann Plast Surg, 2012. **68**(2): p. 135-41.

9. Mailey, B., et al., *Clinical accuracy and reproducibility of Portrait 3D Surgical Simulation Platform in breast augmentation.* Aesthet Surg J, 2013. **33**(1): p. 84-92.

10. Patete, P., et al., *Comparative assessment of 3D surface scanning systems in breast plastic and reconstructive surgery.* Surg Innov, 2013. **20**(5): p. 509-15.

11. Eder, M., et al., *3-D analysis of breast morphology changes after inverted T-scar and vertical-scar reduction mammaplasty over 12 months.* J Plast Reconstr Aesthet Surg, 2013. **66**(6): p. 776-86.

12. Chae, M.P., et al., *3D volumetric analysis for planning breast reconstructive surgery.* Breast Cancer Res Treat, 2014. **146**(2): p. 457-60.

13. Hoeffelin, H., et al., *A methodological evaluation of volumetric measurement techniques including three-dimensional imaging in breast surgery.* Biomed Res Int, 2014. **2014**: p. 573249.

14. Ji, K., et al., *A prospective study of breast dynamic morphological changes after dual-plane augmentation mammaplasty with 3D scanning technique.* PLoS One, 2014. **9**(3): p. e93010.

15. Chen, X. and J. Wang, *Breast volume measurement by mesh projection method based on 3D point cloud data.* International Journal of Clothing Science and Technology, 2015. **27**(2): p. 221-236.

16. Pöhlmann, S.T.L.H., J. ; Williamson, A.I. ; Sergeant, J.C. ; Hufton, A. ; Gandhi, A. ; Taylor, C.J. ; Astley, S.M, *Breast Volume Measurement Using a Games Console Input Device.* IWDM 2014, 2014.

17. Reece, G.P., et al., *3D surface imaging of the human female torso in upright to supine positions.* Med Eng Phys, 2015. **37**(4): p. 375-83.

18. Lacher, R.M., et al., *A comparative study of breast surface reconstruction for aesthetic outcome assessment*. 2017. p. 514-522.

19. Ramsay, J., et al., *Assessment of Breast Asymmetry in Adolescent Idiopathic Scoliosis Using an Automated 3D Body Surface Measurement Technique.* Spine Deform, 2017. **5**(3): p. 152-158.

20. Koban, K.C., et al., *Chances and limitations of a low-cost mobile 3D scanner for breast imaging in comparison to an established 3D photogrammetric system.* J Plast Reconstr Aesthet Surg, 2018. **71**(10): p. 1417-1423.

21. Wesselius, T.S., et al., *Accuracy of Three Software Applications for Breast Volume Calculations from Three-Dimensional Surface Images.* Plast Reconstr Surg, 2018. **142**(4): p. 858-865.

22. Overschmidt, B., et al., *A Prospective Evaluation of Three-Dimensional Image Simulation: Patient-Reported Outcomes and Mammometrics in Primary Breast Augmentation.* Plast Reconstr Surg, 2018. **142**(2): p. 133e-144e.

23. Hummelink, S., et al., *Applications and limitations of using patient-specific 3D printed molds in autologous breast reconstruction.* Eur J Plast Surg, 2018. **41**(5): p. 571-576.

24. Koban, K.C., et al., *3D Mammometric Changes in the Treatment of Idiopathic Gynecomastia.* Aesthetic Plast Surg, 2019. **43**(3): p. 616-624.

25. Catanuto, G., et al., *Breast Shape Analysis With Curvature Estimates and Principal Component Analysis for Cosmetic and Reconstructive Breast Surgery.* Aesthet Surg J, 2019. **39**(2): p. 164-173.

26. Wang, C., et al., *The Effect of Respiration on Breast Measurement Using Three-dimensional Breast Imaging.* Aesthetic Plast Surg, 2019. **43**(1): p. 53-58.

27. McGhee, D.E., et al., *Bra band size measurements derived from three-dimensional scans are not accurate in women with large, ptotic breasts.* Ergonomics, 2018. **61**(3): p. 464-472.

28. Kovacs, L., et al., *Three-dimensional evaluation of breast augmentation and the influence of anatomic and round implants on operative breast shape changes.* Aesthetic Plast Surg, 2012. **36**(4): p. 879-87.

29. Coltman, C.E., D.E. McGhee, and J.R. Steele, *Three-dimensional scanning in women with large, ptotic breasts: implications for bra cup sizing and design.* Ergonomics, 2017. **60**(3): p. 439-445.

30. Howes, B.H., et al., *Magnetic Resonance Imaging Versus 3-Dimensional Laser Scanning for Breast Volume Assessment After Breast Reconstruction.* Ann Plast Surg, 2017. **78**(4): p. 455-459.

31. Pohlmann, S.T.L., et al., *Preoperative implant selection for unilateral breast reconstruction using 3D imaging with the Microsoft Kinect sensor.* J Plast Reconstr Aesthet Surg, 2017. **70**(8): p. 1059-1067.

32. Henseler, H., et al., *Validation of the Kinect device as a new portable imaging system for three-dimensional breast assessment.* J Plast Reconstr Aesthet Surg, 2014. **67**(4): p. 483-8.

33. Wheat, J.S., S. Choppin, and A. Goyal, *Development and assessment of a Microsoft Kinect based system for imaging the breast in three dimensions.* Med Eng Phys, 2014. **36**(6): p. 732-8.

34. Eder, M., et al., *Three-dimensional evaluation of breast contour and volume changes following subpectoral augmentation mammaplasty over 6 months.* Journal of Plastic, Reconstructive & Aesthetic Surgery, 2011. **64**(9): p. 1152-1160.

35. Ruiz, G., et al., *Weighted regularized statistical shape space projection for breast 3D model reconstruction.* Med Image Anal, 2018. **47**: p. 164-179.

36. O'Connell, R.L., et al., *Validation of the Vectra XT three-dimensional imaging system for measuring breast volume and symmetry following oncological reconstruction.* Breast Cancer Res Treat, 2018. **171**(2): p. 391-398.

37. Lee, W.Y., et al., *Three-Dimensional Surface Imaging is an Effective Tool for Measuring Breast Volume: A Validation Study.* Arch Plast Surg, 2016. **43**(5): p. 430-7.

38. Esme, D.L., A. Bucksch, and W.H. Beekman, *Three-dimensional laser imaging as a valuable tool for specifying changes in breast shape after augmentation mammaplasty.* Aesthetic Plast Surg, 2009. **33**(2): p. 191-5.

39. de Runz, A., et al., *Three-dimensional imaging, an important factor of decision in breast augmentation.* Ann Chir Plast Esthet, 2018. **63**(2): p. 134-139.

40. Oren M. Tepper, M., Nolan S. Karp, MD, Kevin Small, BA, Jacob Unger, BA, and B. Lauren Rudolph, Ashley Pritchard, BA, and Mihye Choi, MD, *Three-dimensional imaging provides valuable clinical data to aid in unilateral tissue expander-implant breast reconstruction.pdf.* Breast Cancer Research And Treatment, 2007  **Vol.106** p. pp.S239-S239.

41. Oranges, C.M., et al., *Three-dimensional Assessment of the Breast: Validation of a Novel, Simple and Inexpensive Scanning Process.* In Vivo, 2019. **33**(3): p. 839-842.

42. O'Connell, R.L., et al., *The potential role of three-dimensional surface imaging as a tool to evaluate aesthetic outcome after Breast Conserving Therapy (BCT).* Breast Cancer Res Treat, 2017. **164**(2): p. 385-393.

43. Henseler, H., et al., *The Kinect Recording System for objective three- and four-dimensional breast assessment with image overlays.* J Plast Reconstr Aesthet Surg, 2016. **69**(2): p. e27-34.

44. Henseler, H., et al., *The importance of the pose in three-dimensional imaging of the ptotic breast.* J Plast Reconstr Aesthet Surg, 2013. **66**(11): p. 1551-6.

45. Nahabedian, M.Y. and G. Galdino, *Symmetrical breast reconstruction: is there a role for three-dimensional digital photography?* Plast Reconstr Surg, 2003. **112**(6): p. 1582-90.

46. Henseler, H., et al., *Subjective versus objective assessment of breast reconstruction.* J Plast Reconstr Aesthet Surg, 2013. **66**(5): p. 634-9.

47. Small, K.H., et al., *Re-defining pseudoptosis from a 3D perspective after short scar-medial pedicle reduction mammaplasty.* J Plast Reconstr Aesthet Surg, 2010. **63**(2): p. 346-53.

48. Isogai, N., et al., *Quantitative analysis of the reconstructed breast using a 3-dimensional laser light scanner.* Ann Plast Surg, 2006. **56**(3): p. 237-42.

49. Szychta, P., et al., *Preoperative implant selection for two stage breast reconstruction with 3D imaging.* Comput Biol Med, 2014. **44**: p. 136-43.

50. Henseler, H., et al., *Objective evaluation of the latissimus dorsi flap for breast reconstruction using three-dimensional imaging.* J Plast Reconstr Aesthet Surg, 2012. **65**(9): p. 1209-15.

51. Eder, M., et al., *Objective breast symmetry evaluation using 3-D surface imaging.* Breast, 2012. **21**(2): p. 152-8.

52. Tong, O.L., et al., *Non-contact imaging of breast surface for breast surgical planning*. SPIE BiOS. Vol. 10856. 2019: SPIE.

53. Kovacs, L., et al., *New aspects of breast volume measurement using 3-dimensional surface imaging.* Ann Plast Surg, 2006. **57**(6): p. 602-10.

54. Hummelink, S., et al., *An innovative method of planning and displaying flap volume in DIEP flap breast reconstructions.* J Plast Reconstr Aesthet Surg, 2017. **70**(7): p. 871-875.

55. Ju, X., et al., *Multi-view stereophotogrammetry for post-mastectomy breast reconstruction.* Med Biol Eng Comput, 2016. **54**(2-3): p. 475-84.

56. Veitch, D., et al., *Measurement of breast volume using body scan technology(computer-aided anthropometry).* Work, 2012. **41 Suppl 1**: p. 4038-45.

57. Cohen, O., et al., *Is Unilateral Implant or Autologous Breast Reconstruction Better in Obtaining Breast Symmetry?* Breast J, 2016. **22**(1): p. 75-82.

58. Henseler, H., et al., *Investigation into variation and errors of a three-dimensional breast imaging system using multiple stereo cameras.* J Plast Reconstr Aesthet Surg, 2012. **65**(12): p. e332-7.

59. Tanabe, Y.N., et al., *Intraoperative application of three-dimensional imaging for breast surgery.* Scand J Plast Reconstr Surg Hand Surg, 2005. **39**(6): p. 349-52.

60. Eriksen, C., et al., *Evaluation of volume and shape of breasts: comparison between traditional and three-dimensional techniques.* J Plast Surg Hand Surg, 2011. **45**(1): p. 14-22.

61. Chae, M.P., et al., *Enhancing breast projection in autologous reconstruction using the St Andrew's coning technique and 3D volumetric analysis.* Gland Surg, 2017. **6**(6): p. 706-714.

62. Liu, C., et al., *Does respiration influence breast volumetric change measurement with the three-dimensional scanning technique?* Aesthetic Plast Surg, 2014. **38**(1): p. 115-119.

63. Li, D., et al., *Computation of breast ptosis from 3D surface scans of the female torso.* Comput Biol Med, 2016. **78**: p. 18-28.

64. Santiago, L., et al., *The role of three-dimensional printing in the surgical management of breast cancer.* J Surg Oncol, 2019. **120**(6): p. 897-902.

65. Gladilin, E., et al., *Customized planning of augmentation mammaplasty with silicon implants using three-dimensional optical body scans and biomechanical modeling of soft tissue outcome.* Aesthetic Plast Surg, 2011. **35**(4): p. 494-501.

66. de Heras Ciechomski, P., et al., *Development and implementation of a web-enabled 3D consultation tool for breast augmentation surgery based on 3D-image reconstruction of 2D pictures.* J Med Internet Res, 2012. **14**(1): p. e21.

67. Vorstenbosch, J. and A. Islur, *Correlation of Prediction and Actual Outcome of Three-Dimensional Simulation in Breast Augmentation Using a Cloud-Based Program.* Aesthetic Plast Surg, 2017. **41**(3): p. 481-490.

68. Fujii, T., et al., *Accurate assessment of breast volume by computed tomography using three-dimensional imaging device.* Am Surg, 2012. **78**(9): p. 933-5.

69. Tomita, K., et al., *DIEP Flap Breast Reconstruction Using 3-dimensional Surface Imaging and a Printed Mold.* Plast Reconstr Surg Glob Open, 2015. **3**(3): p. e316.

70. Tomita, K., et al., *DIEP Flap Breast Reconstruction in Patients with Breast Ptosis: 2-Stage Reconstruction Using 3-Dimensional Surface Imaging and a Printed Mold.* Plast Reconstr Surg Glob Open, 2017. **5**(10): p. e1511.

71. Chen, L., et al., *What Makes a Difference? Three-Dimensional Morphological Study of Parameters that Determine Breast Aesthetics.* Aesthetic Plast Surg, 2020. **44**(2): p. 315-322.

72. Schulz-Wendtland, R., et al., *Semi-automated delineation of breast cancer tumors and subsequent materialization using three-dimensional printing (rapid prototyping).* J Surg Oncol, 2017. **115**(3): p. 238-242.

73. Badal, A., M. Clark, and B. Ghammraoui, *Reproducing two-dimensional mammograms with three-dimensional printed phantoms.* J Med Imaging (Bellingham), 2018. **5**(3): p. 033501.

74. Chen, K., et al., *Preoperative breast volume evaluation of one-stage immediate breast reconstruction using three-dimensional surface imaging and a printed mold.* J Chin Med Assoc, 2019. **82**(9): p. 732-739.

75. Catanuto, G., et al., *New technologies for the assessment of breast surgical outcomes.* Aesthet Surg J, 2009. **29**(6): p. 505-8.

76. Ko, B.S., et al., *MRI-based 3D-printed surgical guides for breast cancer patients who received neoadjuvant chemotherapy.* Sci Rep, 2019. **9**(1): p. 11991.

77. Wang, C., et al., *Menstrual Cycle-Related Fluctuations in Breast Volume Measured Using Three-Dimensional Imaging: Implications for Volumetric Evaluation in Breast Augmentation.* Aesthetic Plast Surg, 2019. **43**(1): p. 1-6.

78. Liu, C., et al., *Measuring volumetric change after augmentation mammaplasty using a three-dimensional scanning technique: an innovative method.* Aesthetic Plast Surg, 2012. **36**(5): p. 1134-9.

79. Eriksen, C., et al., *A prospective randomized study comparing two different expander approaches in implant-based breast reconstruction: one stage versus two stages.* Plast Reconstr Surg, 2012. **130**(2): p. 254e-264e.

80. Barth, R.J., Jr., et al., *A Patient-Specific 3D-Printed Form Accurately Transfers Supine MRI-Derived Tumor Localization Information to Guide Breast-Conserving Surgery.* Ann Surg Oncol, 2017. **24**(10): p. 2950-2956.

81. Steen, K., et al., *Three-Dimensional Imaging and Breast Measurements: How Predictable Are We?* Aesthet Surg J, 2018. **38**(6): p. 616-622.
